# Microfinance as a method of facilitating research in emergency medicine

**DOI:** 10.1186/1757-7241-18-23

**Published:** 2010-04-22

**Authors:** Peter Hallas, Mikkel Brabrand, Lars Folkestad

**Affiliations:** 1 The Danish Society for Emergency Medicine, c/o The Emergency Department, Slagelse Hospital, Ingemannsvej 18. 4200 Slagelse, Denmark

## Abstract

Microgrants are a novel concept where small grants are used to facilitate research. The concept might have a place in developing emergency medicine research, especially in countries where emergency medicine is not established or in new areas of research. Two examples of the beneficial effects of microgrants in emergency medicine research are described. Emergency medicine interest groups and foundations should consider setting up microgrant schemes.

## 

Microfinance (small loans for minor projects) has been successfully used for developing small businesses in developing countries for several years. Microfinance in the form of "microgrants" has been suggested as a way of facilitating research projects [1]. In this article we discuss the possibilities of using microgrants to facilitate research in emergency medicine and illustrate the concept with two examples.

## The concept of microgrants in facilitating research

Small research projects with minor funding needs are often met with overwhelming administrative requirements when competing for resources. This obstacle can be difficult to overcome, especially for new researchers with little or no support from more experienced colleagues.

The concept of "microgrants" is to award small grants to researchers who otherwise would have no access to funding [1]. Like microfinanance has been used to develop local business projects in developing countries, research projects suitable for microgrants should focus on local, applied health questions. The projects could have immediate benefit for local health and might in the long run lead to a lasting local tradition of research. Microgrant initiatives should include support from experienced fellow researchers if needed.

Researchers can provide ingenious and innovative approaches to biomedical research and laboratory diagnosis even in the context of very limited resources [2,3]. Limited resources might even create "a creative pressure" that forces scientists to come up with new procedures and research methods [2]. However, the concept of microgrants has been met with worries that the broader impact of research from microgrants projects might be limited because of the local focus of the projects [4].

## Why use microfinance in emergency medicine research?

In most countries emergency medicine is still an overlooked field of research. In Europe some countries (i.e. Denmark) do not even have emergency medicine established as a speciality and many are only beginning to establish an academic tradition in the field [5]. With academic traditions only in their earliest development, there are often limited funding opportunities and few possibilities of support from mentors. Thus, some countries can be considered to be on a "developing level" as far as emergency medicine is concerned, and it might be prudent to use the same method to facilitate research in these countries as in developing countries.

Moreover, few countries have charities that focus primarily on topics in emergency medicine. Charities and foundations often focus on clearly defined diagnostic entities (e.g. "allergy" or "cardiac disease") and patients in the emergency department do not always fit into these categories. Similarly, projects that concern undiagnosed patients or emergency medicine management issues can therefore have difficulties securing major funding. A new concept for facilitating research could possibly help projects focusing on these types of issues. Microgrants could be one of the ways to solve such problems.

## Example 1

### Microgrant in research uncovering organisational issues

The organisation of emergency care in Denmark has been debated for years, but many aspects of how emergency care is organised have not been documented.

We used a microgrant of c. 100 US$ (500 Danish kroner) to perform a questionnaire about acute admissions to Danish hospitals.

The grant (provided by the Danish Society for Emergency Medicine) was used for a gift certificate that participants could win. Distribution of the questionnaire and collection of the data was done on-line literally without any costs.

The project has so far resulted in three publications all pointing out areas where the quality of care can be improved:

- Inexperienced doctors routinely treat critically ill patients with little or no supervision [6].

- There is a gap between guidelines and current practice regarding interhospital transfers [7].

- Medical admission units in Denmark rarely use triage-systems [8].

In addition, there has been a significant local impact of the results with all three articles generating considerable media attention and political discussions. After the publication of preliminary results from [7], a Danish charity has donated 75,000 US$ to an educational programme in interhospital transfers.

## Example 2

### Microgrants in the study of rare procedures

Some life-saving procedures are rarely performed in a normal emergency department, e.g. emergency tracheotomy or intraosseous access. Therefore, only a few doctors get substantial experience with the procedures and even multicenter studies would have difficulties accumulating enough data. This makes it difficult to estimate complication rates and to analyse options for improvement of design of the devices used.

We e-mailed a questionnaire to emergency medicine physicians, anaesthesiologists and paediatricians in Scandinavia about their experiences with intraosseous access.

The budget of c.100 US$ (500 Danish kroner) was used for a gift certificate that participants could win. Distribution of the questionnaire and collection of the data, however, was done on-line literally without any costs.

This project has resulted in the accumulation of detailed data on 2995 attempts to place intraosseous cannulas from a total of 732 Scandinavian health care workers. Analysis of the data is pending. Having data on so many attempts of intraosseous access, is seems unprecedented in the medical literature. We hope that the data can provide us with clues on how to improve the design of intraosseous cannula devices and how adherence to guidelines can be improved.

## Perspectives

In both examples a very small amount of money was used to facilitate important research projects. The grants were used for gift certificates because monetary incentives combined with mail and telephone is known to increase response rates of questionnaires [9]. We are convinced that the small grants were crucial for the projects.

Possibly microfinance in the form of microgrants like these could have a similar impact on other small research projects. Sometimes only a minor amount is sufficient to get a project going. The money might be essential for buying modest equipments, renting manikins, printing booklets, paying publishing fees or (as in our cases) as incentive for respondents of questionnaires.

Most traditional foundations do not bother to provide very small grants or require extensive administrative work regardless of the amount asked for. In addition, it may be hard to convince institutional donors to offer microgrants because of the low profile of many of the projects suited for microgrants [2]. Nonetheless, it could be worthwhile for established foundations to consider microgrants as an option where you get comparatively high value-for-money. Most grant portions might have to be around 1,000 -2,000 US$. But as can be seen from our examples, as little as 100 $ could have a substantial effect as well. National emergency medical societies are recommended also to consider setting up microfinance programmes of their own. This would enable them to target microgrants towards specific areas of interest.

Peer support is an essential component of facilitating research, and microgrant initiatives could therefore include offers of support from fellow researchers [1,10,11].

## Conclusion

Microfinance in the form of microgrants can be used to facilitate research in emergency medicine. The concept could be especially important in countries where emergency medicine is not yet fully developed and for research in areas that do not receive much attention from established foundations. Emergency medicine interest groups, e.g. national societies, should consider setting up programmes for microgrants.
